# The relationship between body image and nutritional behaviors in adult individuals

**DOI:** 10.1371/journal.pone.0320408

**Published:** 2025-03-20

**Authors:** Merve Pehlivan, Neslişah Denkçi, Reyhan Pehlivan, Muhammet Ali Çakır, Yeliz Mercan

**Affiliations:** 1 Department of Nutrition and Dietetics, Faculty of Health Sciences, Trakya University, Edirne, Türkiye; 2 General Directorate of Child Services, Ministry of Family and Social Services, Psychologist, Ankara, Türkiye; 3 Department of Nutrition and Dietetics, Faculty of Health Sciences, Kırklareli University, Kırklareli, Türkiye; 4 Department of Health Management, Faculty of Health Sciences, Kırklareli University, Kırklareli, Türkiye; European University of Rome, ITALY

## Abstract

**Objective:**

Dissatisfaction with body image and maladaptive nutritional behaviors can have profound effects on psychological, social, and physical health and may pave the way for the development of eating disorders. However, research into this topic in the adult population is relatively limited. Therefore, this study aimed to examine various factors affecting dissatisfaction with body image and maladaptive nutritional behaviors in adults living in Türkiye and the relationship between these two concepts.

**Methods:**

This descriptive study was conducted with 3,153 adult individuals who were ≥  18 years old living in Türkiye. The data of the study, which was conducted as an online survey, were collected using the Descriptive Information Form, the Body Image Scale, and the Three-Factor Eating Questionnaire.

**Results:**

Of the participants, 70.1% were women. The mean age was 28.02 ± 9.27 (Min.: 18, Max.: 74) years. The relationship between the mean scores on the total Body Image Scale and Uncontrolled Eating (r = -0.094, p < 0.000), Emotional Eating (r = -0.171, p < 0.001), and Susceptibility to Hunger (r = -0.108, p < 0.001) scores was negative. A statistically significant and positive relationship was detected between the mean scores on the total Body Image Scale and the Cognitive Restraint score (r = 0.089, p < 0.001). Statistically significant relationships were detected in the model adjusted for age and gender between the Body Image Scale and Emotional Eating scores (B = -1.085, p < 0.000), and Cognitive Restraint scores indicated positive relationships (B = 0.848, p < 0.001).

**Conclusion:**

Body image satisfaction was found to be negatively associated with uncontrolled eating, emotional eating, and susceptibility to hunger. On the other hand, a positive relationship was found between body image satisfaction and cognitive restraint. These findings highlight the critical importance of body image satisfaction on eating behaviors and provide potential insight into prevention and intervention programs to improve body image to promote adaptive eating behaviors in the adult population.

## Introduction

Body image has been conceptualized as a subjective mental schema of an individual’s body appearance and functioning [1]. It has been defined as a complex, multidimensional, and dynamic structure that encompasses self-perception and attitudes, including positive and negative emotions, thoughts, beliefs, and behaviors related to the body [2–4]. It is known that body image can be affected by biological (such as gender, age, body weight, and health status), sociocultural (such as social and cultural norms, media influence, and education level), and lifestyle factors (such as physical activity, nutrition, and stress) [2,4,5–7]. In addition, this structure is not static; it is sensitive to aging, health changes, and evolution in the social understanding of beauty [4]. Although body image disturbances have many dimensions, the most frequently addressed concept that represents the attitudinal aspect is body dissatisfaction [1], which refers to negative feelings, thoughts, and evaluations about one’s body (such as body size, shape, muscle tone, and weight) [8]. This situation usually manifests itself as a result of the perceived discrepancy between an individual’s actual body image and ideal body image [6]. Negative body image and body dissatisfaction can profoundly affect individuals’ social relationships, psychological well-being, and eating behaviors [4]. Considering the social and health burden caused by these problems, examination of the factors that lead to body dissatisfaction is of critical importance [9].

Another structure that has a complex nature like body image is nutritional behavior [10–12]. Nutritional behavior can be defined in terms of motivations, such as appetite, hunger, satiety and fullness, the amount and type of food consumed, diet pattern, energy, nutrient profile, and frequency and timing of eating [13]. Beyond biological needs, this behavior can be affected in a complex way by internal and external factors (i.e., physiological, genetic, environmental, lifestyle, emotional, and psycho-sociocultural elements) [10–13]. In this context, eating behavior can be adaptive and maladaptive [14]. Adaptive eating behaviors are shaped by hunger and satiety signals. However, maladaptive behaviors, which develop as a result of disregarding these feelings and as a response to external cues, are associated with unhealthy attitudes and behaviors toward food [14,15]. Three main dimensions of maladaptive eating behaviors have generally been defined in the literature as cognitive restraint, uncontrolled eating, and emotional eating [14,15]. Cognitive restraint is defined as an individual’s conscious effort to limit food intake out of concern for controlling body weight and shape [16,17]. Uncontrolled eating is defined as eating more than normal/overeating in response to external food cues or due to loss of cognitive control over food intake [17,18]. Uncontrolled eating when hungry can be defined as susceptibility to hunger [16]. Emotional eating is often described as overeating in response to negative emotions [17,18]. Such nutritional behaviors may be associated with negative outcomes, such as malnutrition, significant weight gain over time, and the risk of developing eating disorders [19]. Therefore, identifying the potential factors underlying these behaviors is of critical importance.

It is well known that body dissatisfaction is associated with unhealthy weight control behaviors, disordered eating behaviors, and maladaptive eating styles [14,19–24] and is a risk factor for the onset of eating disorders [25,26]. This can be explained in part by sociocultural theories. It is suggested that body dissatisfaction stems from social pressures (e.g., media, family, and peers) that impose unrealistic and often unattainable body ideals. Such social pressures may encourage individuals to adopt these ideals and subsequently change their bodies to fit these norms [9]. In this context, body dissatisfaction and increased negative effects may lead to the emergence of maladaptive nutritional behaviors. These behaviors can be seen as a means of achieving weight control and an ideal body image, and a coping mechanism for negative emotional states, and may pave the way for eating disorders [14,20,27,28].

Considering the negative effects of body dissatisfaction and maladaptive nutritional behaviors, it is clear that it is necessary to examine the relationships between these two concepts and various factors that affect them. However, the majority of the literature has focused on adolescents, young individuals, women [7], and individuals living in Western countries [29]. Therefore, more research is needed on adult populations in non-Western countries [29]. In light of this information, we aimed to investigate the various factors affecting body dissatisfaction and maladaptive nutritional behaviors and the relationship between these two concepts in a large sample of adults living in Türkiye.

## Methods

### Study design

A descriptive design was employed in the present research, which was carried out between May and June 2024 in Türkiye. According to the 2023 data of the Turkish Statistical Institute (TURKSTAT), Türkiye’s population was 85,372,377, and 80.6% of it was aged ≥ 18 [30,31]. The study population was 67,060,744 adults aged ≥ 18 who lived in Türkiye. The inclusion criteria for the study were living in Türkiye, being 18 years of age or older, having internet access, being literate, being able to answer all questions completely, and volunteering to participate in the study. Those who did not meet the inclusion criteria were excluded. The minimum sample size was calculated as 2,383 subjects on the G * Power 3.1.9.4 software to achieve an effect size of d = 0.10, based on a statistical power of 99% and a significance level of α=0.01 [32,33]. Considering some attrition, it was planned to increase the sample size by 30% to reach a total of 3,098 people, and the study was completed with 3,153 individuals.

### Ethical considerations

The study was conducted following the Declaration of Helsinki Principles. To carry out the research, the approval of the Kırklareli University Rectorate Scientific Research and Publication Ethics Board was obtained (15.04.2024-E35523585-199-119823). The written consent of all participants was obtained via Google Forms before the survey was initiated.

### Tools and methods of data collection

All questions asked to the participants were converted into an online survey via Google Forms. Participants were included in the study using the snowball sampling method. The online survey prepared by the researchers was sent to the participants via various social media platforms, such as WhatsApp, Instagram, Facebook, etc. Existing participants were asked to share the survey on their social networks to recruit new participants. Those who read this form and agreed to participate in the study were included in the study. Those who did not approve of this form were not included in the study and could not see the study questions. The Descriptive Information Form, the Body Image Scale, and the Three-Factor Eating Questionnaire were employed as data collection measures.

### Descriptive information form

This form consisted of questions on individuals’ sociodemographic characteristics, health status, and nutritional behaviors. The age variable was divided into two categories as under 25 years of age and those 25 years of age and over, since the mean age was 28 and the median value was 24. The Body Mass Index (BMI) classification of the WHO was utilized (<18.5 kg/m² =  underweight; 18.5-24.9 kg/m² =  normal weight; 25-29.9 kg/m² =  overweight; and ≥ 30 kg/m² as =  obese) [34].

### The three-factor eating questionnaire (TFEQ)

The three-factor eating questionnaire (TFEQ) is a self-report instrument used to assess the four dimensions of nutritional behaviors (uncontrolled eating, emotional eating, cognitive restraint, and susceptibility to hunger). Stunkard and Messick (1985) created this scale with 51 questions [35]. Karlsson et al. (2000) reduced it to 18 items (TFEQ-R18) [36]. Kıraç et al. (2015) performed its Turkish reliability and validity study. The scale has a four-point Likert-type scoring system and four sub-dimensions (uncontrolled eating (questions 7, 13, 14, and 17), emotional eating (questions 3, 6, and 10), cognitive restraint (questions 2, 11, 12, 15, 16, and 18), and susceptibility to hunger (questions 4, 5, 8, and 9) [16]. Questions 1-13 on the scale are scored as follows: definitely correct (4); mostly correct (3); mostly wrong (2) and definitely wrong (1). Questions 14-18 are reverse-scored from 1 to 4. The range of scores on the sub-dimensions is as follows: 5 to 20 points on the uncontrolled eating subscale; 3 to 12 points on the emotional eating subscale; 6 to 24 points on the cognitive restraint subscale; and 4 to 16 points on susceptibility to hunger subscale. High scores show high levels of uncontrolled eating, emotional eating, cognitive restraint, and susceptibility to hunger. The scores obtained were converted to ensure standardization, considering the studies conducted [(The score of the item- possible minimum score)/score range) x 100]. The resulting score is directly proportional to nutritional behavior [16,35,36]. Kıraç et al. (2015) found Cronbach’s alpha value of the total scale score as 0.721 [16]. In the present study, it was calculated as 0.784. The Cronbach alpha value of the subscales of the study in uncontrolled eating was 0.651, emotional eating was 0.877, cognitive restraint was 0.656, and susceptibility to hunger was 0.845. This showed that the reliability of the scale was at an acceptable level [37].

### Body image scale (BIS)

Secard and Jourard (1953) created this scale [38], and Hovardaoğlu (1992) performed its Turkish reliability and validity study [39]. The scale is used to measure how satisfied people are with various parts of their bodies and various body functions. It has one single-factor and scored in the 5-point Likert style. It consists of 40 items, each of which describes an organ or part of the body (such as arms, legs, and face) or a function (such as sexual activity level). They are scored using “1-I do not like it at all,” “2-I do not like it,” “3-I am undecided,” “4-I like it,” and “5-I like it a lot.” Each item is scored from 1 to 5, with 1 indicating the lowest status and 5 indicating the highest status. Scores on the scale range between 40 and 200. High scores indicate high levels of satisfaction with one’s body image. Hovardaoğlu (1992) found Cronbach’s alpha of the total scale as 0.91 [39]. It was calculated as 0.955 in the present study. This showed that the reliability of the scale was at an excellent level [37].

### Statistical analysis

Descriptive values, such as numbers (n), percentages (%), mean, standard deviation ( ± SD), and median values, were utilized in analyses. Reliability analysis was used and the results were tested with Cronbach’s alpha value. Normality was examined with the Shapiro-Wilk test. In cases of nonparametric distributions, the means of two independent groups were compared using the Mann-Whitney U test, and the means of three or more independent groups were compared using the Kruskal-Wallis H test. When the difference was found to be significant in three or more independent groups, Tamhane’s T2 test, a post hoc test, was performed to find the source of the difference. In addition, the effect sizes of the Mann-Whitney U test and the Kruskal-Wallis H test, nonparametric tests, were calculated. Cohen’s d index (d_cohen_) and Eta squared (η2) were used in effect size estimations. Cohen’s d index is interpreted as follows: d = 0.20, small effect; d = 0.50, medium effect; d = 0.80, large effect; and d ≥ 1 very large effect. The Eta squared value is interpreted as a small effect for η2 = 0.01, a medium effect for η2 = 0.06, a large effect for η2 = 0.14, and a very large effect for η2 = 0.20 [40].

The Spearman correlation analysis was employed to investigate the relationship between two continuous variables. The *z* transformation was utilized to ensure that the data had normality. Variables with a p-value less than 0.05 were included in the correlation analysis of the models created with the Enter Method and the relationship between TFEQ sub-dimensions and BIS total was investigated. The explanatory power of the models was shown with Adjusted R square (Adj. R^2^). Analyses were performed on the Statistical Package for the Social Sciences 26.0 (SPSS 26.0) statistical software. The level of significance was set at p < 0.05.

## Results

Participants’ descriptive features are given in Table 1. Mean age was 28.02 ± 9.27 (Median: 24, Min: 18, Max: 74) years, 70.1% were women, 80.9% had an associate or undergraduate degree, 14.1% had a chronic disease, 33.6% were overweight or obese, 43.9% consumed three main meals a day, 58.3% skipped meals, lunch was the most skipped meal (63.9%), 16.7% of the participants consumed three or more snacks, 77.5% consumed inadequate water (women < 2000 ml, men < 2500 ml), and 70.8% did not exercise regularly.

**Table 1:** The distribution of participants’ descriptive characteristics (N = 3153).

Variables	n	%
**Gender**		
Female	2,211	70.1
Male	942	29.9
**Age**		
< 25	1,678	53.2
≥ 25	1,475	46.8
**Education**		
High school and below	601	19.1
Associate degree and above	2,552	80.9
**Chronic disease**		
Yes	444	14.1
No	2,709	85.9
**Body mass index (BMI)** (n = 3,125)		
Underweight	242	7.7
Normal weight	1,832	58.6
Overweight	760	24.3
Obese	291	9.3
**Number of main meals**		
< 3	1,640	52.0
3	1,385	43.9
> 3	128	4.1
**Skipping main meals**		
Yes	1,838	58.3
No	1,315	41.7
**Skipped main meal** ^*^		
Breakfast	834	45.0
Lunch	1,186	63.9
Dinner	190	10.2
**Number of snacks**		
0	458	14.5
1	1,040	33.0
2	1,127	35.7
≥ 3	528	16.7
**Water consumption**		
Insufficient	2,443	77.5
Sufficient	710	22.5
**Regular exercise**		
Yes	921	29.2
No	2,232	70.8

* More than one option was marked.

Table 2 shows the comparison between participants’ descriptive features and their mean scores on the BIS. A statistically significant difference (p < 0.05) was found between the mean BIS score and gender (p < 0.001, η^2^ = 0.044, d_cohen_ = 0.431), chronic disease (p < 0.001, η^2^ = 0.011, d_cohen_ = 0.207), skipping main meals (p < 0.001, η^2^ = 0.019, d_cohen_ = 0.278), skipping snacks (p < 0.001, η^2^ = 0.033, d_cohen_ = 0.372), water consumption (p = 0.003, η^2^ = 0.033, d_cohen_ = 0.372), regular exercise (p < 0.001, η^2^ = 0.024, d_cohen_ = 0.317), and BMI (p < 0.001, η^2^ = 0.007, d_cohen_ = 0.168). According to Eta squared and Cohen’s d index, it was determined that these variables had an effect size below the medium level in the total variance in the dependent variable. In addition, the source of the difference was investigated with the post-hoc test for BMI. Accordingly, the BIS total scores of those in the normal BMI category were determined to be statistically and significantly higher than those in the overweight and obese groups (p < 0.01). Differences between the age groups and educational status and the mean BIS score were not statistically significant (p > 0.05).

**Table 2:** The comparison of participants’ descriptive characteristics with the BIS.

Variables	Mean±SD	Test value	p^1^	*η* ^*2*^	d_Cohen_
**Gender**					
Female	147.50 ± 26.42	−11.841	<0.001^**^	0.044	0.431
Male	159.78 ± 26.38				
**Age**					
<25	150.50 ± 26.84	−1.679	0.134	0.001	0.06
≥25	151.94 ± 27.17				
**Education**					
High school and below	153.11 ± 29.21	−2.260	0.066	0.002	0.081
Associate degree and above	150.72 ± 26.43				
**Chronic disease**					
Yes	144.16 ± 28.46	−5.779	<0.001^**^	0.011	0.207
No	152.32 ± 26.58				
**BMI**					
Underweight^a^	148.54 ± 25.95	24.925	<0.001^*^^*^^,^^2^	0.007	0.168
Normal weight^b^	152.31 ± 26.07				
Overweight^c^	151.87 ± 28.40				
Obese^d^	144.26 ± 28.85				
**Skipping main meals**					
Yes	148.06 ± 27.01	−7.739	<0.001^**^	0.019	0.278
No	155.52 ± 26.38				
**Skipping snacks**					
Yes	148.84 ± 26.49	−7.935	<0.001^**^	0.033	0.372
No	157.43 ± 27.37				
**Water consumption**					
Insufficient	150.41 ± 27.25	−2.924	0.003^*^	0.003	0.104
Sufficient	153.79 ± 25.97				
**Regular exercise**					
Yes	157.50 ± 26.60	−8.790	<0.001^**^	0.024	0.317
No	148.56 ± 26.73				

^1^Mann-Whitney U test,

^2^Kruskal-Wallis H test, *p< 0.01, **p < 0.001, Post hoc test Tamhane's T2 test; BMI:BIS total b > c, b > d.

Table 3 shows the comparison between participants’ descriptive features and their mean scores on the sub-dimensions of the TFEQ. Relationships between the mean Uncontrolled Eating subscale score of the TFEQ and age (p < 0.001, η^2^ = 0.006, d_cohen_ = 0.154), and regular exercise status were statistically significant (p = 0.015, η^2^ = 0.002, d_cohen_ = 0.09). Statistically significant relationships were found between the mean Emotional Eating subscale score and gender (p < 0.001, η^2^ = 0.036, d_cohen_ = 0.385), age (p < 0.001, η^2^ = 0.005, d_cohen_ = 0.146), chronic disease (p < 0.001, η^2^ = 0.004, d_cohen_ = 0.119), skipping main meals (p < 0.001, η^2^ = 0.004, d_cohen_ = 0.123), water consumption (p < 0.001, η^2^ = 0.006, d_cohen_ = 0.152), and regular exercise status (p < 0.001, η^2^ = 0.003, d_cohen_ = 0.111). Statistically significant relationships were detected between the mean Cognitive Restraint subscale score and age (p < 0.001, η^2^ = 0.006, d_cohen_ = 0.152), gender (p < 0.001, η^2^ = 0.018, d_cohen_ = 0.274), water consumption (p < 0.001, η^2^ = 0.019, d_cohen_ = 0.279), and regular exercise status (p < 0.001, η^2^ = 0.021, d_cohen_ = 0.294). The relationship between the mean Susceptibility to Hunger subscale score and, age (p = 0.018, η^2^ = 0.002, d_cohen_ = 0.089), skipping main meals (p = 0.032, η^2^ = 0.002, d_cohen_ = 0.081), water consumption (p = 0.008, η^2^ = 0.002, d_cohen_ = 0.099), and regular exercise status (p = 0.004, η^2^ = 0.003, d_cohen_ = 0.112) was significant. According to Eta squared and Cohen’s d index, it was found that these variables had an effect size below the medium level in the total variance in the dependent variable.

**Table 3:** The comparison of participants’ descriptive characteristics with the TFEQ sub-dimension scores.

*Variables*	Uncontrolled Eating					Emotional Eating					Cognitive Restraint					Susceptibility to Hunger				
**Mean±SD**	**Test value**	**p** ^1^	***η*** ^***2***^	**d**_**Cohen**_	**Mean±SD**	**Test value**	**p** ^1^	***η*** ^***2***^	**d**_**Cohen**_	**Mean±SD**	**Test value**	**p** ^1^	***η*** ^***2***^	**d**_**Cohen**_	**Mean±SD**	**Test value**	**p** ^1^	***η*** ^***2***^	**d**_**Cohen**_
**Gender**																				
Female	11.33 ± 3.31	−0.816	0.557	0	0.029	6.98 ± 2.99	−10.736	<0.001 ^***^	0.036	0.385	14.33 ± 3.55	−4.261	<0.001 ^***^	0.006	0.152	8.56 ± 3.31	−0.245	0.485	0	0.009
Male	11.41 ± 3.38					5.75 ± 2.82					13.76 ± 3.55					8.66 ± 3.57				
**Age**																				
<25	11.63 ± 3.35	−4.325	<0.001 ^***^	0.006	0.154	6.82 ± 2.98	−4.125	<0.001 ^***^	0.005	0.146	13.69 ± 3.50	−7.647	<0.001 ^***^	0.018	0.274	8.72 ± 3.36	−2.517	0.018 ^*^	0.002	0.089
≥25	11.05 ± 3.28					6.38 ± 2.98					14.69 ± 3.55					8.44 ± 3.42				
**Education**																				
High school and below	11.34 ± 3.38	−0.168	0.911	0	0.006	6.53 ± 3.03	−0.870	0.410	0	0.031	14.39 ± 3.60	−1.736	0.082	0.001	0.062	8.70 ± 3.65	−0.331	0.418	0	0.012
Associate degree and above	11.36 ± 3.32					6.64 ± 2.98					14.11 ± 3.54					8.57 ± 3.33				
**Chronic disease**																				
Yes	11.44 ± 3.50	−0.620	0.572	0	0.022	7.08 ± 3.10	−3.368	<0.001 ^***^	0.004	0.119	14.44 ± 3.60	−1.642	0.071	0.001	0.058	8.76 ± 3.49	−1.076	0.246	0	0.038
No	11.34 ± 3.30					6.54 ± 2.96					14.11 ± 3.55					8.56 ± 3.37				
**BMI**																				
Underweight^a^	10.64 ± 3.70	63.201	<0.001 ^***^^,^^2^	0.019	0.28	5.46 ± 2.53	102.551	<0.001 ^***^^,^^2^	0.032	0.363	11.90 ± 3.38	121.993	<0.001 ^***^^,^^2^	0.038	0.398	7.45 ± 3.16	111.648	<0.001 ^***^^,^^2^	0.035	0.38
Normal weight^b^	11.15 ± 3.21					6.43 ± 2.91					14.31 ± 3.54					8.29 ± 3.26				
Overweight^c^	11.68 ± 3.34					6.97 ± 3.10					14.59 ± 3.46					9.21 ± 3.54				
Obese^d^	12.51 ± 3.39					7.90 ± 3.01					13.95 ± 3.38					9.89 ± 3.40				
**Skipping main meals**																				
Yes	11.41 ± 3.33	−0.883	0.251	0	0.031	6.78 ± 3.02	−3.474	<0.001 ^***^	0.004	0.123	14.06 ± 3.54	−1.657	0.055	0.001	0.059	8.70 ± 3.37	−2.292	0.032 ^*^	0.002	0.081
No	11.28 ± 3.32					6.39 ± 2.94					14.30 ± 3.58					8.44 ± 3.41				
**Skipping snacks**																				
Yes	11.31 ± 3.28	−1.437	0.186	0.108	0.697	6.65 ± 2.96	−1.402	0.250	0.109	0.699	14.16 ± 3.48	−0.796	0.929	0.118	0.731	8.59 ± 3.33	−0.616	0.928	0.121	0.741
No	11.49 ± 3.44					6.52 ± 3.07					14.15 ± 3.75					8.58 ± 3.56				
**Water consumption**																				
Insufficient	11.34 ± 3.35	−0.471	0.609	0	0.017	6.49 ± 2.97	−4.306	<0.001 ^***^	0.006	0.152	13.89 ± 3.51	−7.785	<0.001 ^***^	0.019	0.279	8.50 ± 3.39	−2.790	0.008 ^**^	0.002	0.099
Sufficient	11.41 ± 3.27					7.05 ± 3.03					15.09 ± 3.56					8.89 ± 3.39				
**Regular exercise**																				
Yes	11.13 ± 3.30	−2.524	0.015 ^*^	0.002	0.09	6.35 ± 2.93	−3.148	0.001 ^**^	0.003	0.111	15.01 ± 3.69	−8.203	<0.001 ^***^	0.021	0.294	8.32 ± 3.42	−3.153	0.004 ^**^	0.003	0.112
No	11.45 ± 3.34					6.73 ± 3.01					13.81 ± 3.44					8.70 ± 3.37				

^1^Mann-Whitney U test,

^2^Kruskal-Wallis H test,

* p < 0.05,

** p < 0.01,

*** p < 0.001. Post hoc test Tamhane’s T2 test; BMI: Uncontrolled Eating d > a, d > b, d > c, c > a, c > b. Emotional Eating d > a, d > b, d > c, c > a, c > b, b > a. Cognitive Restraint b > a, c > a, d > a, d > c. Susceptibility to Hunger d > a, d > b, d > c, c > a, c > b, b > a.

In addition, a relationship was found between BMI and TFEQ sub-dimension scores (p < 0.001, η^2^ < 0.038, d_cohen_ < 0.398). According to Eta squared and Cohen’s d index, BMI had a below-medium effect size in the total variance in TFEQ sub-dimension scores. The source of the difference was investigated with the post-hoc test. Accordingly, when the TFEQ sub-dimension was examined, uncontrolled eating scores of those in the obese group were determined to be statistically and significantly higher than those in the underweight, normal, and overweight groups. Uncontrolled eating scores of those in the overweight group were determined to be statistically and significantly higher than those in the underweight and normal groups (p < 0.001). Emotional eating scores of those in the obese group were determined to be statistically and significantly higher than those in the underweight, normal, and overweight groups. Emotional eating scores of those in the normal group were determined to be statistically and significantly higher than those in the underweight group (p < 0.01). Cognitive restraint scores of those in the underweight group were found to be statistically and significantly lower than those in the normal, overweight, and obese groups; of those in the overweight group compared to the obese group (p < 0.05). Susceptibility to hunger scores of those in the obese group were found to be statistically and significantly higher than those in the underweight, normal, and overweight groups; of those in the overweight group compared to those in the underweight and normal groups; of those in the normal group compared to those in the underweight group (p < 0.05).

Table 4 shows the associations between the BIS and the sub-dimensions of the TFEQ. The relationship between the mean scores on the total BIS and uncontrolled eating (r =  -0.094, p < 0.001), emotional eating (r =  -0.171, p < 0.001), and susceptibility to hunger (r =  -0.108, p < 0.001) was negative. A statistically significant positive relationship was detected between the mean scores on the total BIS and the cognitive restraint subscale score (r =  0.089, p < 0.001).

**Table 4:** The relationship between the BIS and TFEQ sub-dimension scores.

	Three Factor Eating Questionnaire							
**Uncontrolled Eating**		**Emotional Eating**		**Cognitive Restraint**		**Susceptibility to Hunger**	
**r**	**p**	**r**	**p**	**r**	**p**	**r**	**p**
**BIS**	**−**0.094	<0.001^*^	**−**0.171	<0.001^*^	0.089	<0.001^*^	**−**0.108	<0.001^*^

Spearman Correlation Analysis. r: correlation coefficient. *p < 0.001.

Table 5 shows the multivariate linear regression analysis of the TFEQ sub-dimensions according to participants’ mean BIS scores. In the model adjusted for age and gender, the relationship between the total BIS score and emotional eating (B =  -1.085, p < 0.001) was statistically significant, and a positive relationship was found with cognitive restraint score (B =  0.848, p < 0.001). The relationship between the mean BIS score and uncontrolled eating and susceptibility to hunger subscale scores was not statistically significant (p > 0.05)

**Table 5:** The multivariate linear regression analysis of TFEQ sub-dimensions according to the participants’ total BIS scores.

Predictors	Unadjusted			p	Adjusted^1^			p
	B.	SE	β		B.	SE	β	
**Uncontrolled Eating** ^a^								
BIS total	−0.013	0.002	−0.107	<0.001^*^	−0.182	0.217	−0.022	0.402
**Emotional Eating** ^b^								
BIS total	−0.019	0.002	−0.167	<0.001^*^	−1.085	0.210	−0.120	<0.001^*^
**Cognitive Restraint** ^c^								
BIS total	0.013	0.002	0.097	<0.001^*^	0.848	0.136	0.112	<0.001^*^
**Susceptibility to Hunger** ^d^								
BIS total	−0.013	0.002	−0.105	<0.001^*^	−0.028	0.228	−0.003	0.903

^1^According to age and gender. * p < 0.001

^a^Adj. R^2^: 0.011, F: 36.749

^b^Adj. R^2^: 0.028, F: 90.780

^c^Adj. R^2^: 0.009, F: 30.024

^d^Adj. R^2^: 0.011, F: 34.988

## Discussion

Various components affect body image (e.g., sociodemographic characteristics, body weight, nutritional behaviors, physical activity, and chronic disease). Body dissatisfaction is associated with unhealthy body weight control and eating disorders. This study examined various factors affecting body dissatisfaction and maladaptive eating behaviors and the relationship between these two concepts in a large sample of adult men and women living in Türkiye.

In the current study, women had higher body dissatisfaction than men, similar to the findings of other studies in the literature [2,5,6,41,42]. The higher body dissatisfaction in women than in men may be due to the strict and tight beauty standards imposed by society on the female body. These standards may lead to greater social comparison in women, compared to the more flexible and heterogeneous ones for men [43]. Such norms are often transmitted through peers, family, and the media, and emphasize that physical appearance is more important for women than for men [44]. Pervasive depictions of idealized bodies set unrealistic standards for women and contribute to a social environment in which failure to conform to these standards is often met with criticism and judgment [4]. In such an environment, women are likely to feel greater social pressure to live up to these ideals than men. Although ideals regarding the female body may change over time or according to cultural context (e.g., thin ideal, fit ideal, or thin-thick ideal), it can be said that women are encouraged to change their body and weight to conform to these norms in every period [8]. In this study, gender differences were also found in nutritional behaviors. Women had higher cognitive restraint and emotional eating scores than men, which was consistent with previous studies [12,23,45–50]. These differences are partly attributed to sociocultural norms. Women are exposed to more intense social pressure to achieve an ideal physical appearance or to maintain weight control [45,46]. Therefore, they may have higher body weight concerns than men, which might lead them to be more prone to food restriction to achieve weight control [12]. On the other hand, it has been suggested that men generally focus on increasing muscle mass rather than weight control; therefore, they tend to resort to practices, such as excessive exercise and steroid and supplement consumption instead of dietary restriction [50]. Social pressure toward women and the resulting body dissatisfaction may lead to a more negative mood in women [27]. While women more often turn to eating to cope with negative emotions and stress [12,46], it has been suggested that men prefer alternative ways to cope with such emotional states, such as gambling, alcohol, or internet addiction [51].

This study showed that uncontrolled eating, emotional eating, and susceptibility to hunger were higher in individuals aged < 25 years, while cognitive restraint was higher in those aged ≥ 25 years. Our finding supports the study by Ateş (2022) [52]. In older age, a wider range of emotion regulation strategies [53], reduced levels of anorectic hormones and perception of hunger [54], concerns about health, well-being, and weight control, and ecological and ethical reasons may partly explain these behaviors [55].

This study showed that adults who had chronic diseases had higher body dissatisfaction compared to those who did not.It has been reported that body dissatisfaction is common in individuals with chronic diseases. This may be related to the perception that the disease negatively affects appearance and decreased physical functioning [56,57]. The study also that emotional eating behavior was more in individuals who had chronic diseases compared to those who did not. A limited number of studies in the literature on the investigation of this relationship have shown similar relationships [58,59]. The presence of chronic diseases may trigger emotional eating as a stress factor. On the other hand, emotional eating may pave the way for chronic diseases as it is associated with excessive consumption of unhealthy foods and weight gain [60].

The internalization of the ideal body with the concept of thinness often increases the negative body image [61]. Body image satisfaction was lower in individuals with obesity and overweight in the present study than in individuals with normal weight. In a meta-analysis including 17 articles, adults with obesity reported higher body dissatisfaction than adults with normal weight [42]. Divecha et al. (2022) reported that overweight individuals or those who had obesity had higher body dissatisfaction than those with normal weight. This can be associated with individuals’ social stigmatization associated with overweight [62].

In the current study, it was found that participants who did not exercise regularly had lower body image satisfaction compared to those who did, which was consistent with previous studies [63–65]. More et al. (2019) found that individuals with high body dissatisfaction avoided exercise because they believed it was embarrassing and/or tiring [66]. On the other hand, participation in physical activity can positively affect body perception by improving physical fitness levels [64]. This study showed that cognitive restraint behavior was higher in individuals who exercised regularly compared to those who did not and that uncontrolled eating, emotional eating, and susceptibility to hunger were lower. Although it has been suggested that physical activity may positively affect eating behavior through its positive effects on physiological and psychological factors such as appetite control, food choices, hedonic responses to food stimuli, body image, and self-efficacy, there are conflicting findings in the literature. The complex relationship between physical activity and eating behaviors may vary depending on the type, intensity, and motivation of exercise [67,68].

Body dissatisfaction may be associated with restriction of food intake and disturbed eating behaviors [61,69].The present study indicated that individuals who skipped main meals and snacks had lower body image satisfaction than those who did not, which was consistent with the results of previous studies [70,71].This may be because those who have body dissatisfaction resort to unhealthy weight control behaviors [19]. According to the results of this study, individuals who skipped main meals had higher emotional eating and susceptibility to hunger. Considering that skipping meals is associated with symptoms of mental distress [72], it can be said that this situation may trigger emotional eating behavior. In addition, inadequate energy intake as a result of skipping meals may lead to increased hunger levels, making it difficult to control food intake.

Uncontrolled eating, emotional eating, and susceptibility to hunger scores in the present study were higher in overweight subjects and those who had obesity than in those with normal weight, and cognitive restraint scores was higher in overweight individuals than in those with normal weight. Some studies in the literature have shown relationships between emotional eating and/or uncontrolled eating and overweight/obesity, consistent with our study [73–77]. Emotional eaters prefer sugary and fatty foods, which is associated with an increase in body weight [77]. However, individuals with obesity mostly use emotional eating to cope with stressors such as stigmatization and discrimination [78]. Decision-making mechanisms for self-control of food intake may be impaired in obesity [79]. Evidence on the relationship between restraint behavior and BMI is inconsistent because of the dilemma that this restriction is associated with a healthy nutritional profile and helps with weight control, or on the contrary, causes a desire to eat more and is associated with weight gain [76]. Susceptibility to hunger has been less investigated in the literature, probably because it is affected by acute hunger [80].

As body satisfaction increased, uncontrolled eating, emotional eating, and susceptibility to hunger scores decreased and cognitive restraint score increased in this study. Our finding supports the study by Ateş (2022) [52]. Accordingly, it can be predicted that as people’s body satisfaction increases, they will develop cognitive limiting factors to prevent weight gain by maintaining their body weight. Unlike our findings, some other studies indicated that cognitive limitation increased with a decrease in body satisfaction [61,69]. A previous study showed an association between cognitive restraint and dissatisfaction with the body, and social media pressure on weight loss was shown as a contributing factor to restricted eating [23]. Body dissatisfaction or misperception of body weight is an important reason for restricted eating [23,81]. The role of internalization in the development of human cognitive ability is one of the basic concepts of socio-cultural theory, which argues that individuals behave according to social standards. People will internalize thinness as an ideal and produce a negative body image when their ideal and real images do not match, which will lead to restricted eating behaviors [61]. Studies conducted to increase emotional eating support the results of the present study. Increases in emotional eating scores have been observed along with decreased body satisfaction [20,22,69]. The negative effect caused by body dissatisfaction and the desire to reach the ideal body will increase emotional eating attacks and result in impaired eating behaviors.

### Limitations

There are several limitations to consider when the findings of the current study are interpreted. First, due to the cross-sectional design of the study, it is not possible to make direct inferences about cause-effect relationships. Nutritional behavior and body dissatisfaction in particular may be sensitive to changes over time. Considering the complex structure and interactions of eating behaviors and body dissatisfaction and the factors that affect them, longitudinal studies are needed to examine the dynamics of these relationships over time. Although we used a large sample in our study, the sample consisted only of adults living in Türkiye, which can be considered as a limitation. This limits the generalizability to different age groups, and socioeconomic and cultural groups. Future studies can provide a life-course perspective with larger samples covering different age groups. Considering that Türkiye is located at the intersection of Eastern and Western cultures [82], the results of this study may apply to countries with similar sociocultural dynamics such as the Middle East and the Balkan countries. However, a careful assessment should be made as to whether the results apply to different countries, as well. In particular, it is critical to consider the impact of the Western-centric thin ideal in different cultures. Future studies can make more in-depth comparisons by examining the effects of beauty ideals and body standards on body image and nutritional behaviors in different cultures. BMI, which we used to classify individuals according to body weight in our study, is a common method, but we acknowledge that this measurement has some limitations. Considering only body weight and height in calculating BMI may result in important factors such as body composition and general health being overlooked and may lead to incorrect classifications. In addition, height and weight data obtained through self-reporting may pose a risk of bias. It is recommended that future studies adopt a holistic approach that includes comprehensive measures, such as body composition analysis, physical and mental well-being, and lifestyle factors when the relationship between body image and eating behaviors is evaluated.

## Conclusion

In this study, in which the factors affecting body dissatisfaction and maladaptive nutritional behaviors and the relationship between these two concepts were investigated, a complex scheme of the factors affecting individuals’ body images and eating behaviors was presented, and it was seen that body image had a considerable effect on nutritional behaviors. In addition, considering that body image and nutritional behaviors were affected by different sociodemographic and descriptive data, this relationship was investigated comprehensively by examining its connections with sociocultural and gender dynamics. Considering these complex relationships, conducting community-based intervention and prevention studies will raise individuals’ awareness and prevent body image disorders from leading to eating behavior disorders. When the complex effects of cultural, social, and individual factors on body image are considered, there is an urgent need to develop and implement sound interventions. The establishment of real perception programs regarding individuals’ body perception standards and the implementation of body positivity and self-esteem development programs within educational systems will help to nurture healthy body image from a young age. In addition, countries can promote healthier body image in society by enacting policies that regulate the portrayal of body standards in the media and supporting public health campaigns that accept body diversity. These efforts will contribute to a social framework that supports positive body image and psychological well-being for all individuals and may prevent future processes that may lead to disorders in nutritional behaviors to achieve ideal body perceptions.
